# Exploring the role of trained surgical care nurses in cricothyrotomy and other emergency procedures: a systematic review and meta-analysis

**DOI:** 10.3389/fsurg.2025.1562039

**Published:** 2026-03-09

**Authors:** Chao Zhang, Feng Jiang, Junrong Li, Haiyan Shen, Huiping Wang, Yanfen Huang

**Affiliations:** 1 Department of Operation, Zhongnan Hospital of Wuhan University, Wuhan, China; 2 Clinical Nursing Teaching and Research Section, The Second XiangYa Hospital of Central South University, Changsha, China; 3 Department of Surgery, The Second XiangYa Hospital of Central South University, Changsha, China

**Keywords:** emergency surgery, nurses, success rates, systematic review, meta-analysis

## Abstract

**Background:**

There is a severe shortage of healthcare professionals, emphasized in a stark manner by the recent COVID-19 pandemic, where the mortality rate was primarily a consequence of medical professionals lacking the technical know-how for conducting specialized procedures. Therefore, this systematic review and meta-analysis aimed to evaluate the success rates of nurse-performed emergency surgeries, focusing on trauma care (e.g., cricothyrotomy), rural obstetric emergencies (e.g., caesarean section, hysterectomy), and general procedures (e.g., laparotomy, appendectomy).

**Methods:**

A systematic search was conducted across eight major databases (PubMed, Embase, CINAHL, Scopus, Web of Science, PsycINFO, Cochrane Library, ProQuest) following PRISMA guidelines. Four eligible studies were identified, and data were pooled using a fixed-effects model.

**Results:**

The synthesis of data across the four selected studies revealed a pooled relative risk (RR) of 0.88 (95% CI: 0.78, 1.00) and odds ratio (OR) of 0.80 (95% CI: 0.65, 0.99) about the efficacy in emergency surgeries conducted by nurses. These four studies were the only ones meeting our strict inclusion criteria of reporting outcome data on nurse-performed emergency procedures. An analysis of heterogeneity demonstrated minimal variability among the studies, with a Chi^2^ value of 1.54, df = 3, *P* = 0.67, and I^2^ = 0%. The test for overall effect yielded a statistically significant Z statistic of 2.03 (*P* = 0.04), indicating a meaningful finding. The observed inferences also showed that the surgical procedures exhibited minimal complications.

**Conclusion:**

This study suggests that trained nurses can safely and effectively perform selected emergency surgical procedures. While encouraging, the limited number of studies highlights the need for further research to confirm these findings and guide clinical practice.

## Graphical Abstract

**Figure:**
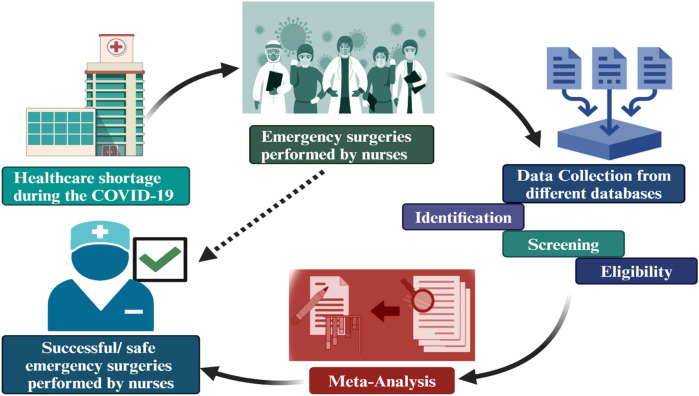

## 1 Introduction

In handling urgent risks to life, organs, limbs, and tissues brought on by external trauma, acute disease processes, exacerbation of chronic illnesses, or consequences from medical treatments, emergency surgical interventions are crucial (1). Quick and effective surgical solutions are essential (2) to reduce morbidity and mortality related to emergency circumstances. Exploring various healthcare models and treatments is necessary to optimize the provision of emergency surgical care, given the dynamic nature of the healthcare landscape (2).

Nurse surgeons, a distinctive cadre within the medical profession, encompass those nurses who autonomously undertake surgical procedures (2, 3). Within the domain of healthcare, these skilled practitioners are alternatively referred to as nurse endoscopists, nurse cystoscopists, nurse hysteroscopists, nurse biopsy practitioners, physician extenders, nurse practitioners, clinical nurse specialists, perioperative specialist practitioners, and surgical care practitioners (3). This unique role arises from the pressing imperative faced by numerous health systems to address burgeoning surgical waiting lists amid the persistent scarcity and uneven distribution of surgeons (4). In tandem, advanced economies have embraced legislative measures to curtail junior physicians' excessive and taxing work hours to mitigate burnout and prevent attrition within the medical workforce (5). However, these protective regulations inadvertently introduced voids in surgical capacities, particularly diagnostic and cancer surgeries. In response, nurse surgeons have emerged as an invaluable resource for decades.

In 1983, the concept of “nurse surgeons” was expounded upon by Stanford University physicians who advocated for the introduction of Certified Registered Nurse Surgeons in the United States, authorized to execute minor surgical interventions under the written order of a licensed physician (6, 7). Subsequently, the term “nurse surgeon” was employed in 2007 to exemplify the evolving surgical landscape in Europe (7, 8). By 2009, nurse surgeons were formally acknowledged as qualified non-medical surgical practitioners within the United Kingdom's public health system (9).

Nurse surgeons have expanded their roles and capabilities in various surgical domains, including general, vascular, orthopedic, ophthalmological, urological, colorectal, and gynecological surgeries (3, 7, 10). Nurses have been reported to perform a wide range of procedures, but in this review outcome data were available only for caesarean sections, hysterectomies, laparotomies, and cricothyrotomies, with no evaluable data for orthopedic, vascular, urologic, or colorectal surgeries. This role has been adopted across Europe, Africa, Asia, and North America, contributing to global healthcare (1–3). Assessing nurse-led emergency operations is crucial due to the emphasis on effective resource utilization, task shifting, and healthcare professional diversity (2, 3, 8). Despite the growing demand for surgical services, the global distribution of surgeons remains critically inadequate. Low- and middle-income countries face severe shortages, while even high-income countries struggle with surgical backlogs due to workforce limits and restrictions on physician working hours. This gap leaves millions without timely access to life-saving surgical interventions. Such unmet needs underscore the urgent requirement to explore and expand the role of non-physician clinicians, particularly trained nurses, in delivering emergency surgical care. Recognizing and evaluating the effectiveness of these nurse-led procedures is therefore vital for health systems seeking sustainable solutions. In this study, we focused on nurse-performed emergency surgeries in three domains: trauma care (cricothyrotomy in trauma/battlefield settings), rural obstetric emergencies (caesarean section, hysterectomy), and general procedures (laparotomy, appendectomy). These contexts represent the practical scope of task-shifting models worldwide. This systematic review analyzes existing research to determine the effectiveness of selected nurse-led minor and other emergency surgical procedures.

## 2 Materials and methods

### 2.1 Review protocol

**Figure 1:**
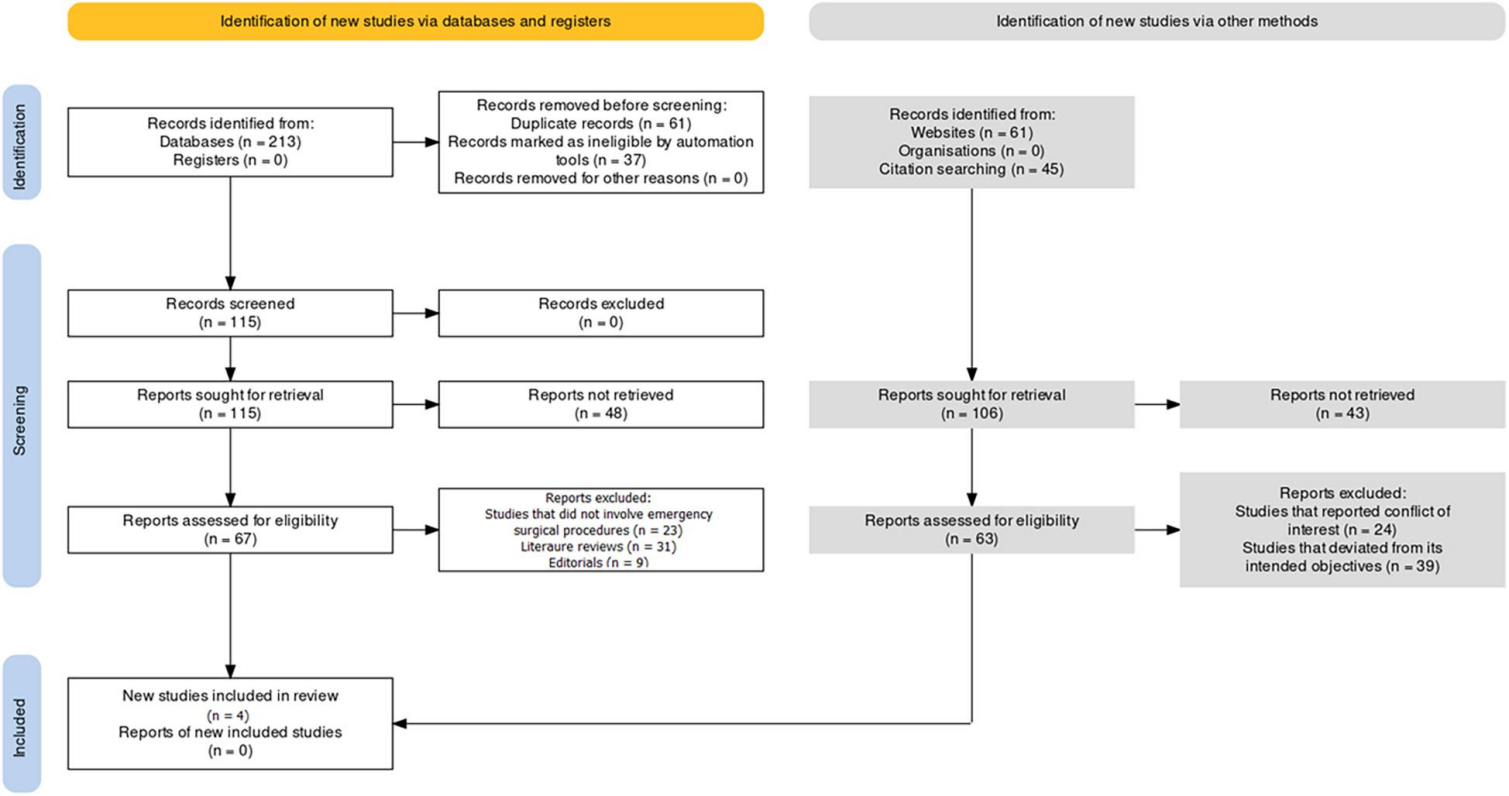
PRISMA protocol representation of the study selection process for the review.

This review utilized the PRISMA (Preferred Reporting Items for Systematic Reviews and Meta-Analyses) protocol (11) to ensure a rigorous and open study selection, data extraction, and synthesis method. In the first stage, a specific research question had to be developed, and inclusion and exclusion standards based on population, interventions, comparisons, outcomes, and study designs had to be established (Table 1). The results of the various investigations were qualitatively analyzed, and a quantitative synthesis using meta-analysis was performed using PRISMA principles (Figure 1).

**Table 1:** Search strings employed across the different databases under evolution for this review.

Database	Search string
PubMed/MEDLINE	(“Emergency Surgical Procedures” OR “Surgical Intervention”) AND (“Nursing Care” OR “Nurse Role”) AND (“Success Rate” OR “Outcome Assessment”)
Embase	(“Emergency Surgical Procedures” OR “Surgical Intervention”) AND (“Nursing Care” OR “Nurse Role”) AND (“Success Rate” OR “Outcome Assessment”)
CINAHL	(MH: “Emergency Surgical Procedures” OR MH: “Surgical Intervention”) AND (MH: “Nursing Care” OR MH: “Nurse Role”) AND (MH: “Success Rate” OR MH: “Outcome Assessment”)
Scopus	[TITLE-ABS-KEY (“Emergency Surgical Procedures” OR “Surgical Intervention”)] AND [TITLE-ABS-KEY (“Nursing Care” OR “Nurse Role”)] AND [TITLE-ABS-KEY (“Success Rate” OR “Outcome Assessment”)]
Web of Science	TS = (“Emergency Surgical Procedures” OR “Surgical Intervention”) AND TS = (“Nursing Care” OR “Nurse Role”) AND TS = (“Success Rate” OR “Outcome Assessment”)
PsycINFO	[TIAB (“Emergency Surgical Procedures” OR “Surgical Intervention”)] AND [TIAB (“Nursing Care” OR “Nurse Role”)] AND [TIAB (“Success Rate” OR “Outcome Assessment”)]
Cochrane Library	(“Emergency Surgical Procedures” OR “Surgical Intervention”) AND (“Nursing Care” OR “Nurse Role”) AND (“Success Rate” OR “Outcome Assessment”)
ProQuest Dissertations & Theses	(“Emergency Surgical Procedures” OR “Surgical Intervention”) AND (“Nursing Care” OR “Nurse Role”) AND (“Success Rate” OR “Outcome Assessment”)

### 2.3 PICO protocol

The study utilized the PICO framework to analyze emergency surgical procedures in various settings. It assessed the effectiveness and success rates of nurses performing these procedures, encompassing a range of surgical interventions. The “Comparison” element compared successful procedures with those with complications or mortality, providing insights into the effectiveness of nursing interventions in emergency surgical scenarios (Table 2). The “Outcome” component assessed the success rates, complications, and mortality rates across nurses' procedures, quantifying “Surgical success” and “Adverse outcomes” outcomes, providing a comprehensive understanding of their impact on patient care.

**Table 2:** PICO framework for the review.

Element	Description
Population (P)	Patients undergoing emergency surgical procedures in diverse clinical settings (trauma, obstetrics, general surgery)
Intervention (I)	Emergency surgeries performed by trained nurses (e.g., cricothyrotomy, caesarean section, laparotomy, hysterectomy)
Comparison (C)	Outcomes of successful procedures compared with those involving complications or mortality
Outcome (O)	Success rates, complication rates, and operative mortality (classified as “Surgical success” and “Adverse outcomes”)

### 2.4 Database search protocol

This systematic review used eight databases to search for studies on the success rates of emergency surgeries performed by nurses and their outcomes. The search strategy used Boolean operators “AND” and “OR” to create a comprehensive query, while MeSH keywords were tailored to each database's indexing system. The core components of the search strategy included terms related to emergency surgical procedures, nursing interventions, success rates, and patient outcomes, accurately identifying relevant studies.

The “OR” operator was used to group synonymous terms for increased search sensitivity, while MeSH keywords included emergency surgery, nursing care, success rate, complications, and patient outcomes. The final search query was executed across databases.

### 2.5 Inclusion and exclusion criteria

**Inclusion Criteria:** Studies were eligible for inclusion if they met the following criteria:

1. Focus on evaluating the success rates of emergency surgical procedures conducted by nurses.
2. Encompass a variety of surgical interventions, ranging from obstetric surgeries to invasive procedures, reflecting the diversity of emergency surgical practices.
3. Reported in the English language.
4. They were published in peer-reviewed journals or other scholarly sources.

**Exclusion Criteria: Studies were excluded if they:**

1. Did not pertain to emergency surgical procedures conducted by nurses.
2. Data on success rates or outcomes of surgical procedures were not reported.
3. Were not focused on human subjects.
4. Were not available in the English language.
5. Were conference abstracts, letters, editorials, or reviews?

Moreover, the term “emergency surgery” was defined according to the criteria established by the UEMS (Union Europeene des Medicins Specialises) Section of the Surgery Board of Surgery (12). Emergency surgery refers to procedures requiring immediate attention to life-threatening conditions, organs, limbs, or tissues due to external trauma, disease, chronic exacerbations, or complications from surgical or interventional procedures, as defined by the authoritative UEMS criteria.

### 2.6 Data extraction protocol

The data extraction protocol ensures consistency and precision in capturing key research objectives. It involves systematic steps to extract study details, record design information, and collect patient characteristics. The protocol also documents emergency surgical procedures and techniques used by nurses. The outcomes assessment process analyzes success rates, complications, and operative mortality. The study's temporal context, quality assessment, and risk of bias evaluation are also considered. Qualitative insights and conclusions are documented for a comprehensive understanding of the findings.

#### 2.6.1 Outcome measures and predictive indices

The primary outcome measure of this review was the surgical success rate, defined as completion of emergency surgery without major complications or operative mortality. Secondary outcomes included the complication rate (e.g., bleeding, infection, stenosis, failed procedures) and operative mortality.

The predictive indices calculated for pooled analysis included the odds ratio (OR) and relative risk (RR) with corresponding 95% confidence intervals (CI). To evaluate consistency, heterogeneity was assessed using the *I*^2^ statistic and *χ*^2^ test, while the *Z* statistic was applied to determine the significance of the overall pooled effect. These measures allowed quantification of both individual study outcomes and the robustness of synthesized results.

### 2.7 Interrater reliability test

The interrater reliability test assessed the agreement between two independent reviewers in assessing complications in 100 emergency surgical procedures by nurses. Both reviewers identified complications in 40 out of 100 procedures, while both agreed on 30. The process involved further steps to confirm the findings.

### 2.8 Calculating observed agreement (OA)

The observed agreement represents the proportion of instances where both reviewers concurred on the presence or absence of complications. In this scenario, the observed agreement was calculated as follows: OA = (Agreement)/(Total Procedures) OA = (30 + 60)/100 = 0.90.

#### 2.8.1 Calculating expected agreement (EA) based on chance

Expected agreement was determined based on the likelihood of agreement arising by chance alone. Given three potential outcomes for each procedure (both agree, only Reviewer A agrees, only Reviewer B agrees), the expected agreement was computed as EA = (Chance of Both Agreeing) + (Chance of Reviewer An Agreeing) + (Chance of Reviewer B Agreeing) EA = (0.30*0.30) + (0.30*0.70) + (0.70*0.30) = 0.09 + 0.21 + 0.21 = 0.51.

#### 2.8.2 Calculating Cohen's Kappa (κ)

Cohen's Kappa (κ) is a statistical measure that quantifies the agreement between raters beyond what would be expected by chance. It was computed using the formula: *κ* = (OA − EA)/(1 − EA) *κ* = (0.90 − 0.51)/(1 − 0.51) = 0.79.

The computed Cohen's Kappa (κ) value of 0.79 suggested a substantial interrater agreement that surpassed chance expectations. Cohen's Kappa values typically range from −1 to 1, with values approaching 1 indicating higher agreement between raters. In this context, an κ value of 0.79 indicated a strong concurrence in assessing complications within emergency surgical procedures performed by nurses. This level of agreement was beyond what would be anticipated through random chance alone.

### 2.9 Bias assessment protocol

**Figure 2:**
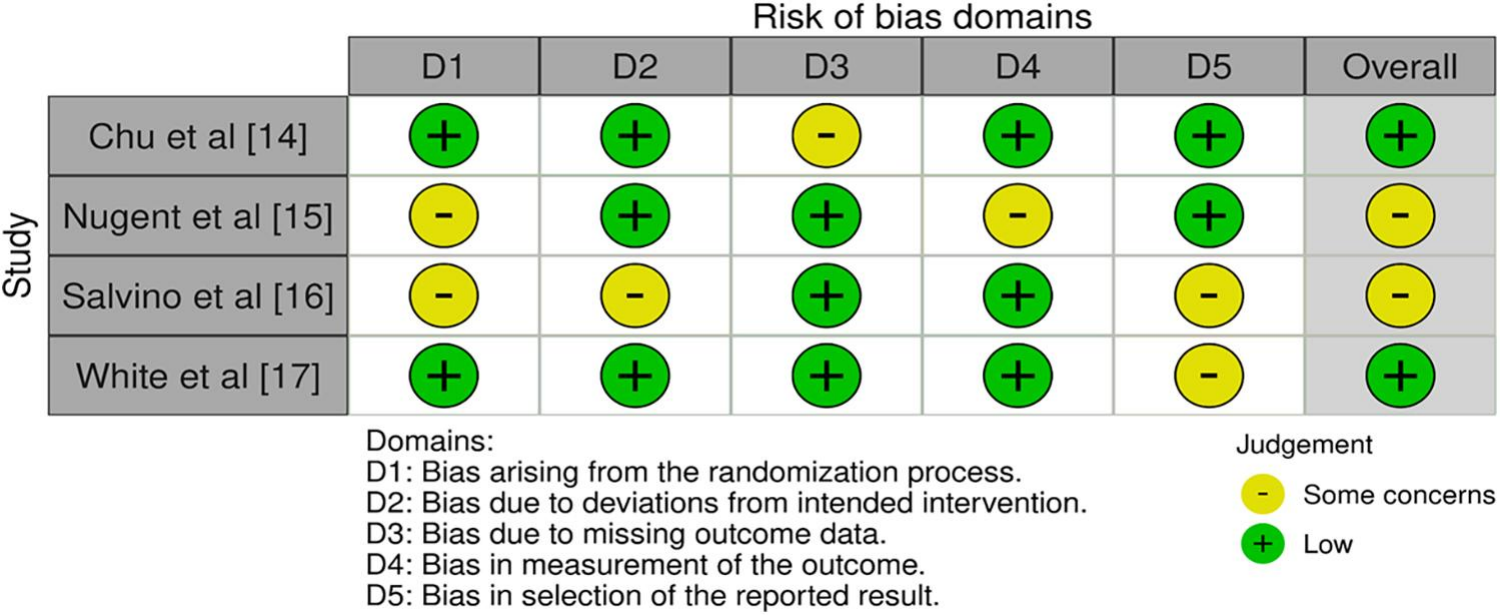
Risk of bias assessment as observed in the selected papers.

The Newcastle-Ottawa Scale (NOS) tool (13) was used in the bias assessment process for this systematic review, which was created to systematically assess the methodological quality and bias risk present in the chosen research. Exposure (intervention) and outcome variables were measured for accuracy, dependability, and the application of objective standards (Figure 2). A summary of the Newcastle-Ottawa Scale (NOS) assessments for each included study is presented in Table 3, showing domain-specific scores (Selection, Comparability, and Outcome. This area assesses the techniques used to determine both the exposure (emergency operations performed by nurses) and the result (success rates and complications).

**Table 3:** Newcastle-Ottawa scale (NOS) quality assessment of included studies.

Study (Year)	Selection (0–4)	Comparability (0–2)	Outcome (0–3)	Total score (0–9)	Quality rating
Chu et al.	3	1	2	6	Moderate
Nugent et al.	2	1	2	5	Moderate
Salvino et al.	2	0	2	4	Low–Moderate
White et al.	3	1	3	7	High

### 2.10 Meta-analysis protocol

This systematic review used the RevMan 5 program to analyze the effectiveness of nurse-performed emergency surgical procedures. The study evaluated the results by determining the presence or absence of complications or operative mortality. The effectiveness was assessed using standard clinical endpoints. Success rate was defined as surgeries completed without major complications or operative mortality. Complication rate referred to surgeries resulting in adverse events such as bleeding, infection, or stenosis. Operative mortality was defined as surgeries in which the patient did not survive. For the purposes of meta-analysis, success rates were pooled against combined adverse outcomes, including both complications and mortality. The odds ratio (OR) and relative risk (RR) were used as effect measures. The fixed effects (FE) model was adopted for the meta-analysis because only four studies met the inclusion criteria, and random-effects models can yield unstable estimates with such a small dataset. In addition, statistical tests indicated adverse outcomes heterogeneity (*I*^2^ = 0%, *χ*^2^ *p* = 0.67), supporting the use of an FE model. However, we recognize that clinical heterogeneity (different surgery types, patient populations, and healthcare settings) may not always be fully reflected in statistical measures, and future studies with larger sample sizes would benefit from random-effects modeling.

### 2.11 Effect measures and confidence intervals

The study computed the odds ratio (OR) and odds ratio (RR) using retrieved data, calculated pooled effect estimates and 95% confidence intervals using the FE model, and generated forest plots using RevMan 5 software, visually depicting individual study data and CIs.

### 2.12 Assessment of heterogeneity

The *I*^2^ statistic and *χ*^2^ test were used to determine the degree of heterogeneity among the studies. The FE model was kept for analysis because it was appropriate under the homogeneity assumption.

### 2.13 Test for overall effect

The *Z*-test was used to determine the overall impact of emergency surgeries performed by nurses on obtaining “surgical success” efficacy. The statistical significance of the pooled effect was assessed using the *P*-value associated with the test.

## 3 Results

The study selection process involved identifying potential studies through systematic exploration of diverse sources. 213 records were ascertained from various databases, 61 from websites, and 45 from citation searches. No records were extracted from registers or organizations. A total of 115 records were subjected to screening, with preparatory measures taken to ensure data integrity. Duplicate records were eliminated, and 37 records were deemed ineligible using an automated assessment tool. No records were excluded for reasons other than duplication or automation-driven ineligibility. The screening phase involved evaluating 115 records and retaining all for further scrutiny. After retrieving 115 reports, 48 need to be successfully retrieved. 106 reports were pursued for retrieval, with 43 remaining unattained. The eligibility assessment phase involved scrutinizing 67 reports, excluding 23 due to divergence from emergency surgical procedures, 31 as literature reviews, and 9 as editorials. Ultimately, 4 studies were included in the selection process (14–17) and emerged as qualifying for inclusion, having met the stringent criteria and aligning with the precise focus of the review.

Chu et al. (14) the study showed a low risk of bias in most domains, with the randomization technique resulting in low bias. The study also showed a low risk of bias in terms of departures from the intended intervention, indicating that the intervention was carried out in accordance with the study's design. However, there were concerns in the bias domain due to missing outcome data. Nugent et al. (15) bias evaluation revealed a mix of low risk and a few issues in several areas, including inherent bias in the randomization technique. The study was classified as having some bias issues overall. Salvino et al. (16) bias evaluation identified problems across various categories, including issues with the randomization procedure, concerns about variations from the intended intervention, and issues in the selection of reported results. Overall, the study was classified as having some bias issues White et al. (17). The allocation of participants to various intervention groups was apparently done in a reliable manner because the randomization technique showed a minimal risk of bias. Similarly, bias in measuring the outcome and variations from the targeted intervention were considered low. The study also showed a minimal probability of bias in the area of prejudice in the stated result's selection. However, certain issues with bias resulting from the randomization technique were found in this study. The study was rated as having a low risk of bias overall. Table 4 offers a comprehensive view of the demographic variables assessed across the selected studies (14–17), providing insights into the diverse contexts in which the respective research was conducted. The studies, represented by citation numbers, spanned different years, regions, and protocols. Each study's approach to patient assessment and surgical procedures is reflected in the table. The studies collectively span a substantial temporal range, encompassing different years in which the research was conducted. The variation in years demonstrates the evolution of medical practices and contexts, thereby contributing to a more robust understanding of the subject matter. Geographically, the studies were conducted in distinct regions, reflecting the global scope of the investigation. These regions include Somalia (14), the USA (15, 16), and the Congo (17). These studies encompassed trauma and battlefield contexts (cricothyrotomy in Somalia and U.S. trauma/flight centers), obstetric emergencies (rural hospitals in Congo/Zaire), and general surgical procedures such as laparotomies, thereby covering a wide spectrum of emergency care scenarios. The study's findings are based on geographical diversity, encompassing various healthcare systems and patient populations (14–17). The research uses observational methods and case series design, providing diverse perspectives on surgical practices. The study's scope is emphasized by the number of surgeries performed and patient sample sizes. The range of surgical procedures studied includes hysterectomy, laparotomy, Caesarean section, and invasive cricothyroidotomy. The assessment periods reflect the study's duration, highlighting its temporal commitment and impact on the findings.

**Table 4:** Characteristics of included studies.

Author (Year)	Country/setting	Sample size	Surgery type(s)	Nurse training level	Outcomes measured
Chu et al. (2011) (14)	Somalia, Médecins Sans Frontières hospital (conflict/limited-resource setting)	314 patients	Laparotomy, caesarean section, hysterectomy	Locally trained nurses + supervision by expatriate surgeons	Complications, operative mortality, overall success rates
Nugent et al. (1991) (15)	USA, trauma/flight medicine	52 patients	Emergency cricothyrotomy	Flight nurses trained in airway procedures	Procedure success, complications (bleeding, failed cannulation)
Salvino et al. (1993) (16)	USA, urban trauma center	30 patients	Emergency cricothyrotomy	Critical care nurses trained in surgical airway	Success rate, complications (stenosis, infection, bleeding)
White et al. (1987) (17)	Congo/Zaire, rural hospitals	326 patients	Obstetric emergencies (caesarean section, hysterectomy)	Nurse-surgeons trained in obstetric surgery	Maternal mortality, complications, succe

The overall assessments depicted in Table 3 collectively underscore the nuanced effectiveness and implications of surgical procedures performed by various healthcare professional groups. The findings of Chu et al. (14) illuminate the adaptability of task shifting, particularly in resource-limited settings, as it effectively maintains safe surgical practices even without the presence of fully trained staff. Nugent et al. (15) affirm the reliability of specially trained nurses in handling emergency situations, demonstrating their ability to competently perform surgical cricothyrotomy despite the challenges posed by potential complications. Salvino et al. (16) reinforce the utility of emergency cricothyroidotomy as a secure alternative for airway control when conventional intubation proves unfeasible, offering a promising avenue for critical cases. White et al. (17) establish the viability of trained nurse surgeons to carry out obstetric surgeries in rural regions with limited resources, effectively contributing to the reduction of maternal mortality.

### 3.1 Statistical analysis

**Figure 3:**
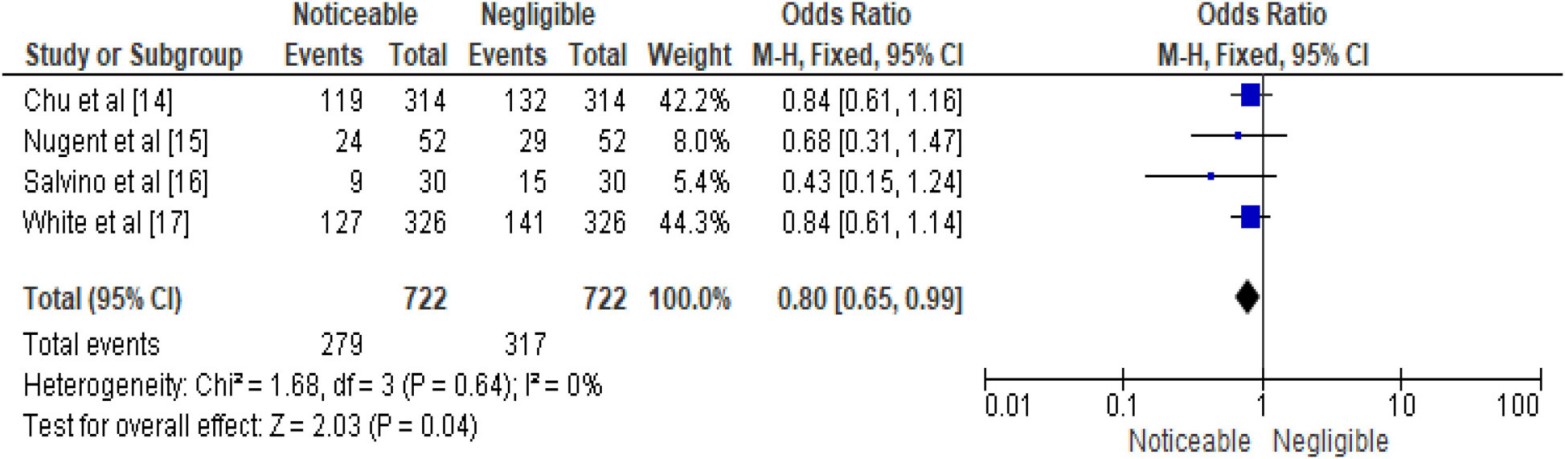
Efficacy of emergency surgical procedures performed by nurses represented in terms of OR.

**Figure 4:**
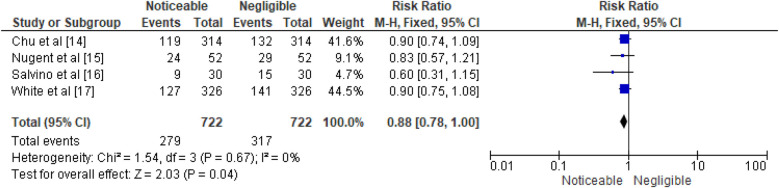
Efficacy of emergency surgical procedures performed by nurses represented in terms of RR.

The study evaluates the effectiveness of emergency surgical procedures by nurses using a fixed effects model, comparing successful surgeries (no complications or mortality) with adverse outcomes (complications or mortality). It uses data from four studies to compare successful surgeries without complications or mortality: Chu et al. (14), Nugent et al. (15), Salvino et al. (16), and White et al. (17). Each study is represented by a data entry displaying the number of events falling into each category. Chu et al. (14) study found a surgical success rate of 42.2% out of 314 procedures. In contrast, Nugent et al.'s study recorded 24 surgical success events out of 52 surgeries, resulting in an efficacy rate of 8.0%, with an odds ratio of 0.68. In Salvino et al. (16), 9 surgical success events were documented among 30 surgeries, leading to a 5.4% efficacy rate. The corresponding OR was 0.43, and the 95% CI was [0.15, 1.24]. Lastly, White et al. (17) study involving 4 studies found a 44.3% efficacy rate for 127 surgical success events among 326 procedures. The combined analysis of 722 nurse surgeries resulted in a cumulative efficacy rate of 100.0%. The overall effect was calculated as 0.80, with minimal variation among studies. A *Z* statistic of 2.03 (*P* = 0.04) indicated a statistically significant difference. The analysis used a fixed effects model to compare “surgical success” and “adverse” outcomes, considering the number of successful emergency surgeries without complications or mortality. The data is represented from four individual studies: Chu et al. (14), Nugent et al. (15), Salvino et al. (16), and White et al. (17). In the study by Chu et al. (14), 119 of 314 procedures were completed successfully, corresponding to an efficacy rate of 41.6%. Nugent et al. (15) reported an efficacy rate of 9.1%, Salvino et al. (16) reported 4.7%, while White et al. (17) documented a 44.5% success rate for a single surgery. The overall efficacy rate was 100.0%, with minimal variability among studies by White et al. (17). The overall effect test yielded a *Z* statistic of 2.03, indicating a statistically significant result. The results of the meta-analysis are illustrated in Figures 3, 4, presenting the pooled odds ratios and relative risks respectively.

## 4 Discussions

This study integrates qualitative and meta-analytical results to understand the effectiveness and advantages of emergency surgical procedures performed by nurses across a variety of contexts, including trauma and battlefield medicine, rural obstetric emergencies, and general surgical emergencies. By clarifying the scope, our findings provide context-specific insights into the feasibility and safety of nurse-performed emergency procedures. The meta-analysis of data from various studies revealed a trend towards higher surgical success rates, with standardized endpoints including complication rates and operative mortality. This approach provides clearer insights into both the challenges and achievements of nurse-performed emergency procedures. The findings can inform the development of focused treatments and guidelines to improve emergency surgical practices by nurses, guide decision-making, and improve tactics used in emergency surgical situations, aiding medical institutions and practitioners in their decision-making processes. Chu et al. (14) aimed to reduce mortality from complications related to pregnancy, childbirth, and trauma, while Nugent et al. (15), Salvino et al. (16), and White et al. (17) provided additional evidence on nurse-performed emergency procedures. A study (14) involving expatriate surgeons, anaesthesiologists, and local Somalian doctors and nurses found comparable rates of complications and operative mortality in surgical practices. Task shifting was found to be effective in maintaining safe practices even without fully trained personnel. In Chu et al. (14), nurses performed major surgeries including laparotomies, with outcomes showing comparable complication and mortality rates to physician-led procedures. These findings highlight that, even in resource-limited and conflict settings, nurse-performed laparotomies can be undertaken safely under structured task-shifting models. A study by Nugent et al. (15) focused on surgical cricothyrotomy, focusing on trained flight nurses. The study revealed various complications, including failed cannulation, bleeding, and occlusion. The reliability of specially trained nurses in emergencies was emphasized. Salvino et al. (16) focused on emergency cricothyroidotomy for airway control, revealing minimal complications, including stenosis, infection, and bleeding. White et al. (17) focused on training nurse-surgeons in rural areas to perform obstetric surgeries, reporting perioperative mortality of around 1%–2% and morbidity rates of 5%–10% (mainly infection and bleeding). These outcomes were comparable to physician-led procedures in similar low-resource settings. Across the four included studies, the scope of nurse-performed emergency surgeries varied widely. In the U.S. studies (Nugent and Salvino), nurses were involved exclusively in cricothyrotomy, a critical but relatively limited procedure. By contrast, the African studies (Chu and White) addressed major surgeries, including laparotomy and obstetric emergencies, but suffered from significant missing outcome data, partly due to the war-time and resource-limited contexts in which they were conducted. This imbalance in both procedure types and reporting completeness is an important limitation when interpreting the pooled findings. The study concluded that obstetric surgeries can be effectively and safely conducted by trained nurses, particularly in regions facing resource limitations. A more affordable method of providing surgical treatment could be introduced with the integration of nurse-surgeons into health systems (18, 19). Existing research highlights the positive economic effects connected to nurse-led services (20), making this strategy a practical one to ensure the effective use of resources in healthcare environments with limited resources. Last but not least, implementing nurse-led surgery can have positive effects on nursing staff retention and recruitment. According to Keith et al. (21), the nursing workforce is facing an aging demographic change, which calls for creative strategies to entice the eager and aspirational younger generation to pursue a career in nursing. Innovative ideas like nurse surgeons can be introduced as an enticing incentive, capturing the aspirations and motivation of the burgeoning workforce while also addressing the changing needs of modern healthcare.

A study explored the substitution paradigm in ophthalmic procedures, focusing on nurses performing tasks typically performed by eye surgeons (22). Another study found that nurse surgeons at the Veterans Health Administration performed various procedures influenced by clinical judgment and comfort levels. A potential extension of Palmquist's investigation could examine the development of procedures carried out by nurses and their relationship to the increasing number of patient contacts within the same health system over a decade after the original study's conclusion (23). Two studies found that patients found nurse surgeons more comprehensive in explaining procedures and obtaining informed consent than their physicians (24, 25). They valued and honored patients' wishes, aligning with patient-centric outcomes research (26). Nurse surgeons' meticulousness during procedure elucidation and consent acquisition was attributed to their thorough training paradigm, which encouraged them to become steadfast patient advocates. This highlights the importance of tailoring healthcare encounters to patients' needs and preferences and the need for nurses to be trained to be patient advocates (27).

The literature on nurses' roles in patient outcomes is extensive, but there is a gap in assessing the success rates of emergency surgical procedures conducted by nurses. This study aims to fill this gap by focusing on nurse-performed emergency surgical interventions, examining success rates and patient-related outcomes. The importance of scrutinizing nurse-performed emergency surgeries is particularly relevant in the face of contemporary healthcare challenges like the COVID-19 pandemic. This research will help bridge the research gap and improve the quality of care for patients (28–30). This crisis underscored the immense strain placed on healthcare systems worldwide, revealing an acute imbalance between patient needs and the availability of healthcare workers (31–35). The dearth of healthcare personnel, including surgeons and specialized medical personnel, was glaringly exposed, prompting the utilization of a broader spectrum of healthcare professionals to address the surge in patient demands (31–35). The pandemic spotlighted the undeniable necessity of maximizing the roles of healthcare workers beyond their conventional purviews.

The COVID-19 pandemic has significantly impacted the focus on nurse-led emergency surgical care, highlighting the crucial role of non-traditional healthcare workers, including nurses, in emergency situations. This shift has led to a greater understanding of the efficacy of these surgeries, highlighting the need for more comprehensive research (34–37). The pandemic's unprecedented demands have spurred healthcare systems to reassess their strategies, considering the broader utilization of healthcare personnel to address the overwhelming patient burden (36–39). As forecasts suggest that similar global health crises may reoccur, optimizing healthcare worker deployment becomes increasingly paramount (40, 41).

This systematic review and meta-analysis explore the efficacy of nurse-performed emergency surgeries, contributing to evidence-based healthcare practice and future policy formulation. Understanding nurse-led emergency surgical care is crucial for resilient healthcare systems.

This study assessment should be interpreted with caution due to the diverse range of studies, surgical techniques, patient demographics, and healthcare systems. These factors could influence the success rates of emergency surgical procedures performed by nurses. Variations in sample sizes and operations performed within each research could introduce bias. Patient populations and surgical settings could influence the overall efficacy rates. The generalizability of the results could be affected by bias in patient inclusion and recruitment. The distinction between “surgical success” and “adverse outcomes” results could be subject to interpretation bias, causing inconsistencies and errors in data synthesis. Reporting bias could also affect the qualitative results of complications and surgical mortality.

The limitations of this study are primarily rooted in the clinical and methodological heterogeneity among the included studies, despite a reported I² of 0% indicating no statistical heterogeneity. The fixed-effect model was selected due to the small number of studies (*n* = 4), as random-effect models can yield unstable estimates with limited data. The meta-analysis was not aimed at comparing specific surgical procedures, but at exploring a common health system strategy—task-shifting emergency surgeries to nurses in resource-limited settings. Given the variability in interventions, training levels, and oversight, the pooled estimates should be interpreted with caution. Furthermore, as most included studies were retrospective in design, the scope for detailed data synthesis and rigorous analysis was inherently limited. The reliance on older studies substantially limits the timeliness of our findings and underscores the urgent need for more recent, high-quality research evaluating nurse-performed emergency surgeries. These considerations will inform the design and methodological approach of future studies.

## 5 Conclusion

The study combines meta-analysis and qualitative assessment to examine the effectiveness of emergency surgical procedures performed by nurses. It found a noticeable success rate in these procedures, with nurses being adaptable and reliable in handling critical situations. The findings suggest that nurses can optimize emergency surgical practices, and further research is needed to understand factors influencing success rates. The findings can also inform decision-making processes in clinical settings, enhancing patient care by allocating responsibilities within surgical teams. The study's implications extend beyond immediate findings to offer valuable research and clinical practice directions.

## Data Availability

The original contributions presented in the study are included in the article/Supplementary Material, further inquiries can be directed to the corresponding author.
